# Go Fish! Hepatic Uptake of Clinical Hepatospecific Gadolinium-Based MRI Contrast Agents in Zebrafish is Similar to Humans

**DOI:** 10.1007/s11307-025-02023-2

**Published:** 2025-06-03

**Authors:** Josie A. Shapiro, Tapas Bhattacharyya, Lauren A. Squire, Christiane L. Mallett, Jeremy M.-L. Hix, Legend E. Kenney, Aitor Aguirre, Erik M. Shapiro

**Affiliations:** 1 James Madison College, Michigan State University, East Lansing, MI 48824 USA; 2 Department of Radiology, Michigan State University, East Lansing, MI 48824 USA; 3 Department of Physiology, Michigan State University, East Lansing, MI 48824 USA; 4 Department of Chemical Engineering and Material Science, Michigan State University, East Lansing, MI 48824 USA; 5 Department of Biomedical Engineering, Michigan State University, East Lansing, MI 48824 USA; 6 Institute for Quantitative Health Science and Engineering, Michigan State University, East Lansing, MI 48824 USA

**Keywords:** Zebrafish, MRI, Gadolinium

## Abstract

**Purpose:**

Zebrafish are a useful organism for investigating liver disease due to their genetic similarities with humans, particularly in genes associated with liver function. It has been posited that liver function can be assessed non-invasively by MRI, measuring the hepatic accumulation of gadolinium-based contrast agents (GBCAs). We characterized the hepatic uptake of various hepatospecific and non-hepatospecific clinical GBCAs in zebrafish.

**Procedures:**

To introduce GBCAs systemically, zebrafish swam for 30 min in water containing 5 mM of various clinical hepatospecific or non-hepatospecific GBCAs. Fish were then sacrificed and underwent 3D, T1-weighted, high-resolution MRI at 9.4 T. In vitro MRI and transport studies of the same GBCAs were conducted in HEK293T cells transiently expressing OATP1D1, OATP1B2 and OATP1B3.

**Results:**

T1-weighted ex-vivo MRI of zebrafish showed hyperintensity in the liver, gall bladder, bile ducts, and intestine for fish swimming in gadoxetate, but not for in gadobenate nor gadoterate. In vitro cell experiments confirm that gadoxetate but not gadobenate is efficiently transported by OATP1D1.

**Conclusion:**

Zebrafish liver accumulates gadoxetate but not gadobenate via OATP1D1 transport, among the two clinical hepatospecific MRI GBCAs, and also does not accumulate gadoterate, a non-hepatospecific GBCA. This pattern of GBCA hepatic uptake is similar to humans but differs from all other non-primates reported, which exhibit high hepatic uptake of both gadoxetate and gadobenate.

## Introduction

Liver diseases affect 100’s of millions of people worldwide, accounting for 4% of all deaths globally [1]. Early detection and precise staging of liver diseases can significantly enhance medical outcomes by enabling timely and targeted interventions. Identifying liver diseases at an early stage allows for treatments that can halt or reverse progression, reduce complications, and improve survival rates. Accurate staging informs clinicians about the severity and extent of the disease, guiding appropriate therapeutic decisions—from lifestyle modifications and medications to surgical options like transplantation. This proactive approach not only improves patient quality of life but also reduces healthcare costs associated with managing advanced liver diseases.

Accurately assessing liver function is critical for diagnosing and managing liver diseases such as chronic liver disease (CLD), cirrhosis, hepatocellular carcinoma (HCC), and drug-induced liver injury (DILI). Invasive methods such as liver biopsy, suffer from patient morbidity and sampling variability. Non-invasively derived serum biomarkers such APRI and FIB-4, though widely used, can sometimes be limited in detecting early-stage liver dysfunction [2]. This has led to a growing interest in non-invasive imaging techniques that provide real-time, quantitative assessments of liver function to improve early diagnosis and management [3–5].

An important aspect of liver function is the clearance of exogenously delivered pharmaceuticals from the blood, mediated by active transporter-mediated influx, metabolism in some cases, and transport-mediated excretion into the bile. One or more of these processes can be impaired in multiple liver diseases and are postulated to be differentially impaired in distinct phases and types of disease. One example is metabolic dysfunction in both Type 1 and Type 2 diabetes, where hepatic influx transporters in the OATP1B family are downregulated, leading to the poor clearance of exogenously delivered pharmaceuticals from the blood [6]. Non-invasive MRI methods can harness this phenomenon to quantitatively measure the hepatic influx and efflux of MRI-imageable substrates, such as gadoxetate (Gd-EOB-DTPA), which normally exhibits efficient influx by the OATP1B transporters expressed on the basolateral side of hepatocytes [3]. Since liver dysfunction often manifests as reduced activity of specific hepatic transporters, such as OATP1B’s responsible for basolateral influx, and MRP2 mediating canalicular efflux, quantifying gadoxetate transport dynamics via MRI enables early detection and functional stratification of liver diseases [3, 5, 7, 8].

Many studies have explored gadoxetate-enhanced MRI to detect liver metabolic function in rodent models, as there are many reproducible and meaningful rodent models of acute and chronic liver diseases. However, zebrafish are also a valuable organism for investigating liver diseases, as 69% of human genes have zebrafish orthologs [9]. Zebrafish possess conserved hepatic gene expression networks that mirror those in humans, including key regulators of liver development, lipid metabolism, and regeneration. This genetic similarity enables zebrafish models to accurately recapitulate human liver disease phenotypes, such as steatosis and fibrosis, making them powerful tools for mechanistic studies and drug screening [10]. Further, zebrafish enable rapid and high-throughput experimentation, making them instrumental for drug screening and the identification of potential therapeutic interventions for liver diseases. In this study, we sought to answer two questions. First, can hepatospecific MRI contrast agents enter the fish circulatory system by having fish swim in contrast agent containing water? This was important because, for mammals, systemic delivery is facile, but for fish, an alternative easy method is required. Secondly, which human hepatospecific MRI contrast agents are accumulated in the zebrafish liver? While humans efficiently influx only gadoxetate via OATP1B1 and OATP1B3 [11], rodents efficiently transport both gadoxetate and gadobenate through OATP1B2 (and OATP1A1, for which there is no human ortholog) [12]. Zebrafish also express a hepatic OATP (OATP1D1, 43% identical to OATP1B3), which acts as an influx transporter [13, 14], but its characterization for the transport of MRI contrast agents has not been reported. Herein, we characterized, for the first time, the hepatic uptake of various hepatospecific and non-hepatospecific clinical gadolinium-based contrast agents (GBCAs) in zebrafish and offer insight on the use of zebrafish as a model for imaging liver diseases by MRI.

## Materials and Methods

All animal experiments were carried out under approved animal use protocols approved at Michigan State University. Live zebrafish (AB wildtype, both sexes), *N = *5 for each condition, were ‘incubated’ for 30 min in 5 mM of various clinical GBCAs by diluting GBCAs in water and letting fish swim in tanks. Hepatospecific GBCAs were gadobenate and gadoxetate. Non-hepatospecific GBCA was gadoterate. Control fish swam in water without any GBCA. Fish were then sacrificed by overdose of tricaine in the same GBCA-containing water. Fish underwent brief microCT examination immediately after sacrifice to select fish for MRI. Fish with large air pockets in the digestive system were ruled out owing to the difficulty in imaging the liver with air in the intestine. Individual fish then underwent MRI investigation at 9.4 T (Bruker Biospin) in small plastic tubes, either in air or submerged in water. 3D, T1-weighted, high-resolution gradient echo MRI was performed with the following parameters: TR = 30 ms, TE = 3.5 ms, FA = 50°, 3.00 × 1.28x1.28 cm FOV, 256 × 128x128 acquisition matrix, 1 NEX, total imaging time = 8.2 min. Frequency-selective fat saturation was important, implemented with a narrowband Gaussian 512 pulse centered on the fat peak, with spoiler gradients to dephase saturated fat spins.. Image analysis was performed in AMIDE.

Transient transfections of cells to express various hepatic OATPs were carried out in triplicate by using Calcium Phosphate Transfection Kit (OZ Biosciences, USA). For OATP1D1, a mammalian expression system was designed and ordered from Vectorbuilder, expressing zebrafish SLCO1D1 [NM_001348086.1] with a 6XHis tag, driven by EF1a promoter. Rat OATP1B2 and human OATP1B3 plasmids were lentiviral expression plasmids designed and ordered from Vectorbuilder with rat SLCO1B2 [NP_113838.1] or human SLCO1B3 [NP_062818.1] expression driven by EF1a promoters. Each well of 6-well plates was used for individual transfections. One day before the transfection (Day 0), 5 × 10^5^ HEK293T cells were seeded in each well of 6-well plates with 2 ml of cell culture media (DMEM with 10% FBS). On the day of transfection (Day 1), old media was replaced by 2 ml of fresh media and incubated at 37 °C in CO_2_ for 2 to 4 h before the transfection. For each transfection, 4 µg of plasmid DNA was used, along with 120 µl of 1 × HBS (HEPES Buffer Saline) and 6.6 µl of CaCl_2_ (2.5 M). Control cells were mock-transfected. The next day (Day 2), the old media with the transfection reagents was replaced by fresh, prewarmed media. On the next day (Day 3), the uptake assays were carried out. Transiently transfected cells were incubated with 2.5 mM of individual MRI GBCAs for 1 h in growth media. Cell pellets were washed several times with PBS and imaged via MRI (see below). After MRI, cell pellets were dehydrated and acid digested, and ICP-MS (Agilent 8900 QQQ-ICP-MS) was used to measure gadolinium content.

T1 maps of cell pellets were acquired on a Bruker 70/30 MRI with Paravision 360 v3.5. Cell pellets in 300 µL tubes were imaged, 12 at a time, in the center of an 86 mm transmit/receive volume coil. Imaging parameters were: T1 map_RARE sequence, RARE factor = 2, TR = (5000, 1439, 760, 360, 74) ms, TE = 7 ms, 1 average, 1 mm sagittal slices 60 × 40 mm with 240 × 160 matrix for 250 × 250 voxel size, scan time = 9 min. T1 values were calculated in Paravision using the image sequence analysis module.

## Results

Our preliminary set of studies had fish swimming in GBCA-containing water, removal to fresh water for sacrifice, then MRI. This study revealed several pitfalls. First, no signal enhancement from GBCA accumulation was evident anywhere in the fish, suggesting that if there was some accumulation, the time it took animals to succumb might be long enough for the GBCA to leave the body. A second observation was the presence of air bubbles in the gastrointestinal (GI) system of some of the fish, which created susceptibility artifacts in the gradient echo MRI. Attempts to use spin echo methods to mitigate the air bubble artifacts produced unsatisfactory images in the brief acquisition period desired. A third observation was the susceptibility artifact from the air-filled swim bladder, precluding our ability to image the kidneys that are proximal to this structure. Lastly, rapid deterioration of the fish once sacrificed was evident, requiring fast image acquisition. Given this, we settled on a new scheme where fish were sacrificed in GBCA-containing water, and after sacrifice, fish underwent a quick microCT prior to MRI to rule out fish with GI air bubbles.

**Fig. 1:**
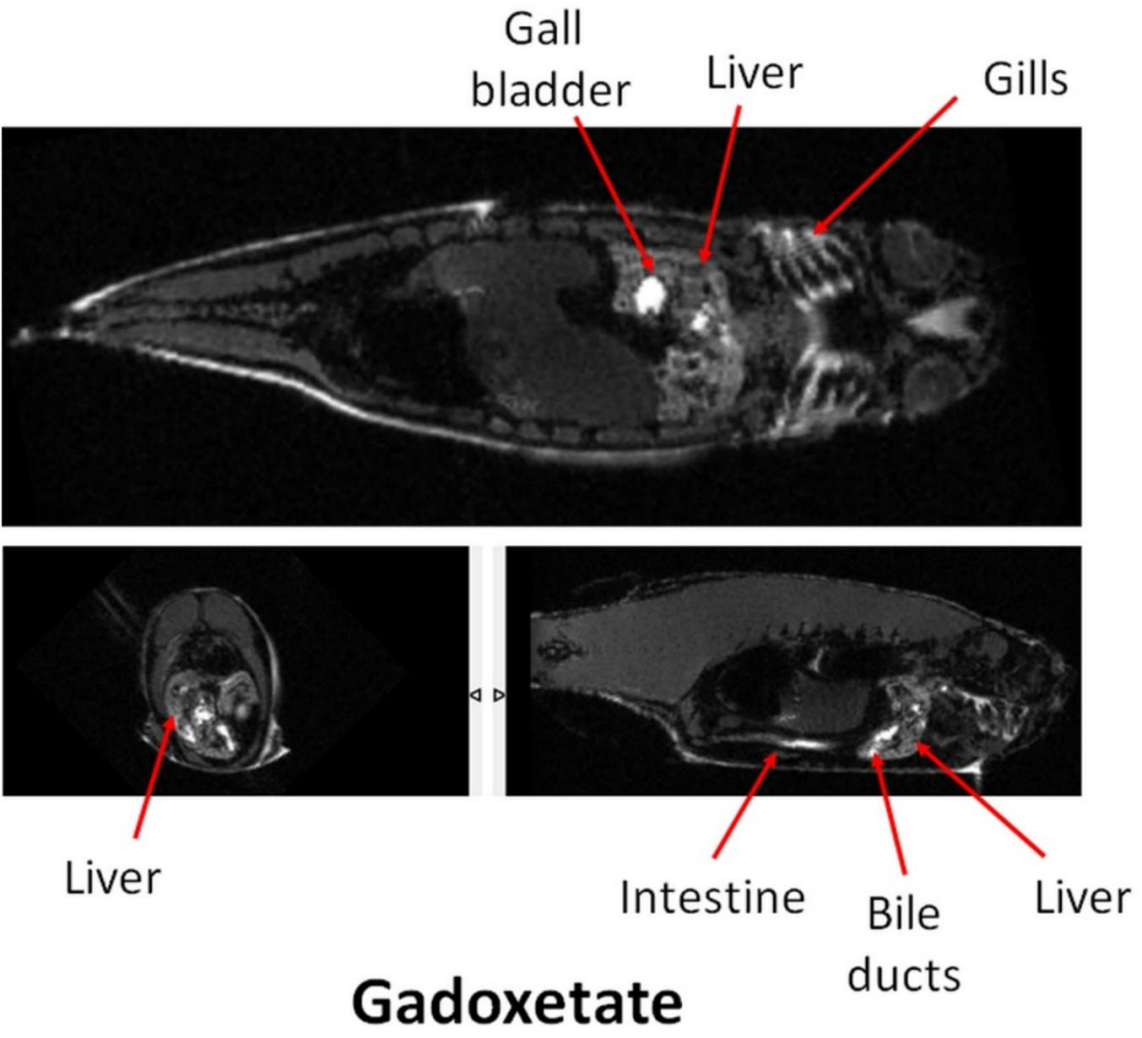
Orthogonal MRI slices of a zebrafish that swam in water containing gadoxetate. Red arrows point to anatomical locations as indicated. In addition to the gills, the full hepatobiliary system is hyperintense, including the liver, gall bladder, bile ducts and intestine

Figure 1 shows three orthogonal MRI slices from a fish that swam in gadoxetate containing water. The gills are immediately recognizable as hyperintense, as is the liver and gall bladder. Indeed, the gall bladder and several vessel-like structures, more easily observed when panning through images, are very hyperintense, suggesting high accumulation of gadoxetate in bile via hepatic metabolism. The intestine was hyperintense, demonstrating a similar hepatobiliary excretion route to humans.

**Fig. 2:**
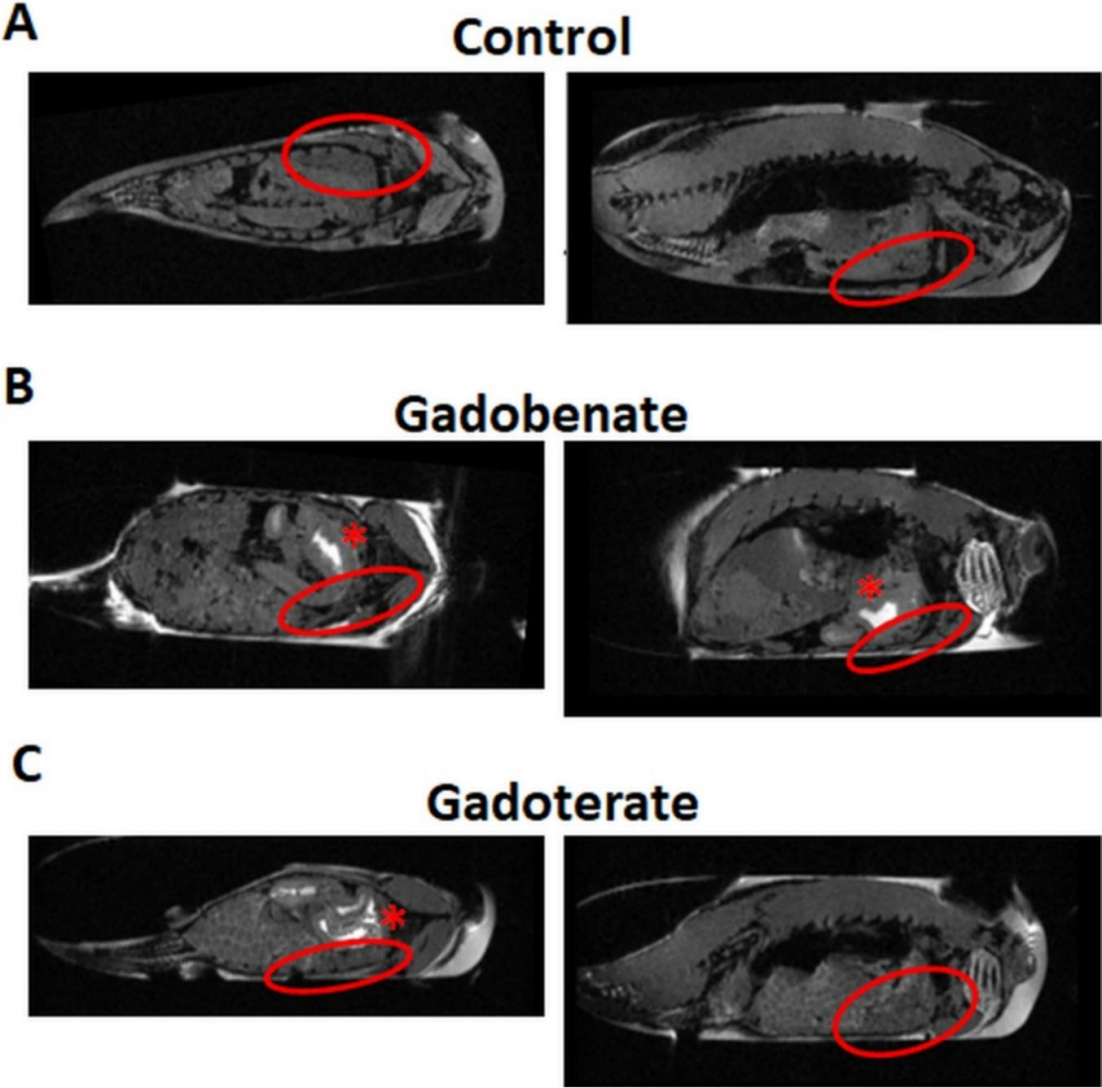
Orthogonal MRI slices from 3 different fish that swam in water containing (**A**) nothing, (**B**) gadobenate or (**C**) gadoterate. No parts of the hepatobiliary system are hyperintense in any of these animals. The liver is circled in red, and the hyperintense signal in the stomach is indicated by a *

Figure 2 shows MRI slices of fish that swam in plain water, gadobenate or gadoterate. Control fish show even signal intensity throughout the body (Fig. 2A). MRI of fish who swam in gadobenate containing water showed no hyperintensity in liver, bile vessels or intestine, only in the stomach and gills (Fig. 2B). The same was observed for fish that swam in gadoterate containing water (Fig. 2C). All animals within cohorts (*N* ≥ 3) exhibited similar imaging findings, but with some variance in hepatic signal enhancement in the gadoxetate group, perhaps due to tissue deterioration.

To investigate what role OATP1D1 plays in the hepatobiliary clearance of gadoxetate, we transfected HEK293T cells with a plasmid encoding OATP1D1 and tested for the intracellular accumulation of GBCAs. Additional conditions were cells transfected with plasmids encoding the rat OATP1B2 and human OATP1B3 transporters. Positive controls were cells expressing OATP1B2 incubated with both gadoxetate and gadobenate (efficient transport of these agents) [15], and OATP1B3 incubated with gadoxetate (efficient transport of this agent) [12, 15], while negative controls were OATP1B3 incubated with gadobenate (poor transport of this agent) [15] and untransfected cells incubated with both gadoxetate and gadobenate (no transport of these agents).

**Fig. 3:**
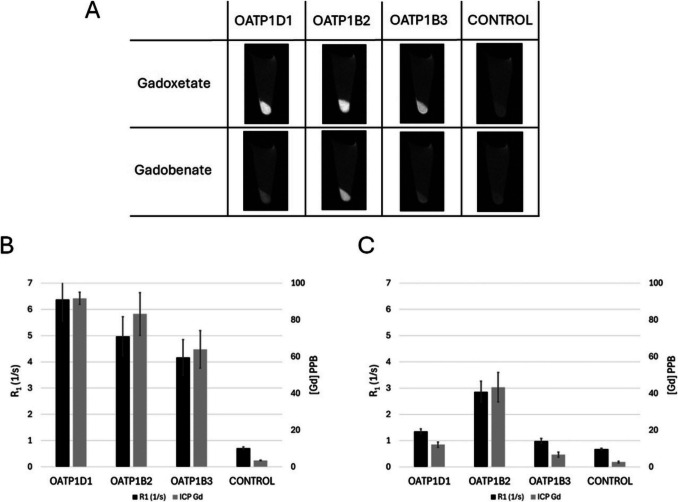
**A** MRI slices from the T1 maps (TR = 360 ms) for cell pellets of cells expressing either OATP1D1, OATP1B2, OATP1B3, or non-expressing, incubated in either gadoxetate or gadobenate. **B** and **C** graph measured R1 rates for cells pellets along with measured gadolinium content for (**B**) gadoexetate and (**C**) gadobenate. Error bars are standard deviations from *N = *3 individual replicates

Figure 3A shows T1-weighted images of cell pellets (TR = 360 ms) after incubation with GBCAs. Pellets from cells expressing each OATP appeared bright following exposure to gadoxetate, while only OATP1B2-expressing cells were bright after incubation with gadobenate. Figures 3B and C present the calculated R1 relaxation rates alongside gadolinium concentrations (measured by ICP-MS) for cells incubated with gadoxetate and gadobenate, respectively. The results from both MRI and ICP-MS confirmed expected transporter-specific uptake. Positive controls (OATP1B2 + gadoxetate/gadobenate, OATP1B3 + gadoxetate) showed high signal and gadolinium content while negative controls (OATP1B3 + gadobenate, and untransfected cells) showed low signal and negligible uptake. Importantly, OATP1D1-expressing cells demonstrated strong uptake of gadoxetate but minimal uptake of gadobenate, closely mirroring the behavior of OATP1B3. These findings confirm that the observed MRI contrast resulted from differential gadolinium accumulation based on transporter specificity. These in vitro results corroborate our in vivo observations, confirming that gadoxetate, but not gadobenate, is selectively transported into the zebrafish liver via OATP1D1, consistent with its hepatic accumulation pattern on MRI.

## Discussion

Our findings demonstrate that zebrafish exhibit a pattern of hepatic uptake for GBCAs that closely mirrors that of humans, especially regarding hepatospecific agents. Specifically, gadoxetate showed strong hepatic uptake and biliary excretion, while gadobenate and gadoterate did not accumulate appreciably in the liver. These results highlight a potentially translational advantage of zebrafish in modeling human liver function using MRI. A key finding in this study was that this hepatic uptake could be achieved by having fish swim in GBCA-containing water, rather than the need for a more invasive method.

The uptake of gadoxetate into the zebrafish liver, evidenced by hyperintense signals in the liver, gall bladder, and intestine, suggests that OATP1D1 (the zebrafish ortholog of human OATP1B3) is a functionally active transporter for this agent. This is further confirmed by our in vitro cell experiments, in which HEK293T cells expressing OATP1D1 efficiently accumulated gadoxetate, but not gadobenate. These results are consistent with prior data on human OATP1B3 and support the notion that zebrafish OATP1D1 behaves more like its human counterpart than its rodent analogs.

Interestingly, this pattern diverges from what has been reported in other non-primate species such as rodents, where both gadoxetate and gadobenate show high hepatic uptake due to the presence of OATP1B2 and OATP1A1, the latter having no human or zebrafish ortholog. Thus, zebrafish may provide a more human-relevant model for studying transporter-mediated hepatic processes than rodents, especially for drugs or imaging agents where OATP specificity is crucial.

However, several limitations in our experimental approach need to be addressed. First, the imaging was performed post-mortem due to technical challenges in live imaging. This prevented us from performing dynamic imaging, which is essential for assessing pharmacokinetics in real time. Additionally, we found that contrast signal could diminish rapidly after sacrifice, possibly due to fast clearance or diffusion, which limits the post-mortem imaging window. While in vivo zebrafish MRI has been accomplished, it is not a simple task, requiring specialized setups [16].

From a translational standpoint, our results suggest that zebrafish could serve as a screening platform for hepatobiliary MRI contrast agents or drugs affected by OATP-mediated transport. Their genetic similarity to humans and ease of high-throughput testing are clear advantages. That said, the anatomical differences and current lack of standard imaging pipelines for live zebrafish MRI pose barriers to broader adoption.

Overall, we found that fish exhibit similar hepatic disposition of GBCAs as humans, but owing to some challenges in imaging live fish and unfamiliarity in their anatomy, their use as a translational imaging tool remains to be defined.

## Data Availability

MRI data sets will be made available to researchers upon reasonable request to EMS.
